# Integrated network findings reveal ubiquitin-specific protease 44 overexpression suppresses tumorigenicity of liver cancer

**DOI:** 10.18632/aging.204733

**Published:** 2023-05-18

**Authors:** Huanhuan Zhou, Lu Yang, Xiao Lin, Ting Fung Chan, Nikki Pui-Yue Lee, William Ka Fai Tse, Xing Zhang, Rong Li, Keng Po Lai

**Affiliations:** 1 Key Laboratory of Environmental Pollution and Integrative Omics, Education Department of Guangxi Zhuang Autonomous Region, Guilin Medical University, Guilin, Guangxi, PR China; 2 Department of Oncology, The Second Affiliated Hospital of Guilin Medical University, Guilin, Guangxi, PR China; 3 Department of Psychiatry, Icahn School of Medicine at Mount Sinai, New York, NY 10029, USA; 4 School of Life Sciences, State Key Laboratory of Agrobiotechnology, The Chinese University of Hong Kong, Hong Kong SAR, China; 5 Department of Surgery, University of Hong Kong, Hong Kong SAR, China; 6 Center for Promotion of International Education and Research, Faculty of Agriculture, Kyushu University, Fukuoka 819-0395, Japan

**Keywords:** tumor microenvironment, cell-cell interactions, hepatocellular carcinoma, USP44, cell proliferation, transcriptome

## Abstract

Hepatocellular carcinoma (HCC) is the sixth most common cancer and third leading cause of cancer-related deaths worldwide. HCC is a multistep disease marked by various signaling alterations. A better understanding of the new molecular drivers of HCC could therefore provide an opportunity to develop effective diagnostic and therapeutic targets. Ubiquitin-specific protease 44 (USP44), a member of the cysteine protease family, has been reported to play a role in many cancer types. However, its contribution to HCC development remains unknown. In the present study, we observed suppression of USP44 expression in HCC tissue. Clinicopathologic analysis further showed that low USP44 expression correlated with poorer survival and a later tumor stage in HCC, suggesting that USP44 could be a predictor of poor prognosis in patients with HCC. Gain-of-function analysis *in vitro* demonstrated the importance of USP44 in HCC cell growth and G_0_/G_1_ cell cycle arrest. To investigate the downstream targets of USP44 and the molecular mechanisms underlying its regulation of cell proliferation in HCC, we conducted a comparative transcriptomic analysis and identified a cluster of proliferation-related genes, including *CCND2*, *CCNG2*, and *SMC3.* Ingenuity Pathway Analysis further delineated the gene networks controlled by USP44 through the regulation of membrane proteins and receptors, enzymes, transcriptional factors, and cyclins involved in the control of cell proliferation, metastasis, and apoptosis in HCC. To summarize, our results highlight, for the first time, the tumor-suppression role of USP44 in HCC and suggest a new prognostic biomarker in this disease.

## INTRODUCTION

Liver cancer is one of the most prevalent human malignancies, ranking third as a leading cause of cancer-related deaths worldwide [1]. In 2020, 905,677 cases of liver cancer were diagnosed globally, with a mortality-to-incidence ratio of 0.6. The main reason for the high mortality rate is the lack of a satisfactory curative pharmacologic treatment. Hepatocellular carcinoma (HCC), comprising 75–85% of cases, is the foremost type of liver cancer. HCC is characterized by active angiogenesis and metastasis, which account for its rapid recurrence and poor survival rates [2]. The need to identify novel biomarkers for diagnosing HCC and therapeutic targets for its treatment is therefore urgent. A number of signaling pathways essential for cell survival, metastasis, and DNA damage response are known to be dysregulated in HCC [3–6]. A better understanding of the regulatory mechanisms underlying HCC carcinogenesis will help in the development of novel molecular therapeutic targets. Deubiquitination (the removal of ubiquitin from proteins) regulates protein degradation by proteasomes and lysosomes. Deubiquitination is controlled by deubiquitinating enzymes that cleave the bonds between ubiquitin and its substrate protein. In humans, more than 100 deubiquitinating enzymes have been identified. Of the five established classes of these enzymes [7], a major one is the ubiquitin-specific proteases (USPs). The functional diversity of these proteases is demonstrated in the profound effect they have on multiple biologic processes through the post-translational regulation of their interacting proteins. Accumulating evidence has demonstrated the importance of the USPs in carcinogenesis [8, 9]. Within this enzyme class, ubiquitin-specific protease 44 (USP44) has been found to be dysregulated in cancers such as colon cancer, leukemia, and glioma [10–12]. *USP44*, a gene located on human chromosome 12, consists of 712 amino acids. It has conserved cysteine, histidine, and aspartate residues, which are signature features of deubiquitinated enzymes, and through its unique ZnF-UBP structural domain, it participates in the regulation of protease activity [13]. A copy number meta-analysis of 12 human cancer types showed that *USP44* is a novel tumor suppressor [14, 15]. USP44 has also been reported to be associated with breast cancer aggressiveness [16]. Currently, however, the abundance and functional role of USP44 in HCC remain unknown. In the present study, we conducted clinicopathologic analysis to investigate the prognostic value of USP44 in HCC. Furthermore, we used HepG2 cells to characterize *USP44* gain-of-function in HCC. Finally, we performed a comparative transcriptomic analysis followed by a bioinformatic analysis, including Ingenuity Pathway Analysis, to understand the molecular mechanisms and signaling pathways underlying the roles of USP44 in HCC tumorigenesis. This report provides evidence that USP44 might be a novel therapeutic target in HCC.

## MATERIALS AND METHODS

### Patient sample collection

Retrospectively collected clinical specimens were used in this study. Consent was obtained from all patients. The patients’ tumors were histologically confirmed as hepatocellular carcinoma. The data were collected from 2007–2008. The Institutional Review Board of the University of Hong Kong/Hospital Authority Hong Kong West Cluster approved the use of the clinical specimens for research. The data were extracted from the electronic patient record system under the IRB reference number (UW 05-359 T/1022). Tissue specimens for RNA extraction were immediately preserved in RNA*later* (Thermo Fisher Scientific, Waltham, MA, USA) and fixed in formalin for preparation of paraffin blocks. Clinical specimens were collected and processed by surgical tissue bank staff in the Department of Surgery, University of Hong Kong. Patients undergoing tumor resection at Queen Mary Hospital were included. The clinicopathologic parameters required for this study were available from the surgical tissue bank.

### cDNA synthesis and quantitative real-time PCR

RNA was isolated from 40 pairs of HCC and adjacent nontumoral liver tissues using TRIzol reagent (Tiangen, Beijing, China) according to the manufacturer’s instructions. First-strand cDNA was synthesized from 1 μg total RNA using the FastKing gDNA Dispelling RT SuperMix Kit (Tiangen). Expression levels of *USP44* were determined using the SuperReal PreMix Plus (SYBR Green) kit for quantitative real-time PCR (RT-PCR). Emission intensity was detected using the CFX96 Touch Real-Time PCR Detection System (Bio-Rad Laboratories, Hercules, CA, USA) and following these steps: initial denaturation step at 95°C for 15 min, and 40 thermal cycling steps consisting of 10 s at 95°C, 20 s at 55°C, and 30 s at 72°C. Threshold cycles were averaged from triplicate reactions. To adjust for variations in the starting template, gene expression was normalized to glyceraldehyde 3-phosphate dehydrogenase. The mRNA expression of *USP44* in HCC was compared with that in adjacent nontumoral liver tissue. The primer sequences were shown below (GAPDH_F: 5′-GAGAAGGCTGGGGCTCATTT-3′; GAPDH_R: 5′-AGTGATGGCATGGACTGTGG-3′; USP44_F: 5′-CCCTCAGACCAGAATGCTTTA-3′; USP44_R: 5′-CATTGCAGTGTACCCAGAACC-3′). For the transcriptome sequencing validation, the primer sequence of the select genes was shown below (CCND2_F: 5′-TTTGCCATGTACCCACCGTC-3′; CCND2_R: 5′-AGGGCATCACAAGTGAGCG-3′; CCNG2_F: 5′-CAGGATTGAGAAATGCCAAAGT-3′; CCNG2_R: 5′-TGACAGCCAGGACAAAAGTT-3′; SMC3_F: 5′-ATTGGTGCCAAAAAGGATCAGT-3′; SMC3_R: 5′-GATTGCTTCGAGAAAAACCAGC-3′; THBS1_F: 5′-GCTGGAAATGTGGTGCTTGTCC-3′; THBS1_R: 5′-CTCCATTGTGGTTGAAGCAGGC-3′; FZD2_F: 5′-GTGCCATCCTATCTCAGCTACA-3′; FZD2_R: 5′-CTGCATGTCTACCAAGTACGTG-3′; ROCK2_F: 5′-TGGTTTCTATGGGCGAGAATGT-3′; ROCK2_R: 5′-CAAGTCGTACCTCCCTATCTGTT-3′).

### Cell culture

The HCC cell line HepG2 was cultured in minimal essential medium (Solarbio Science and Technology, Beijing, China) supplemented with 10% (v/v) fetal bovine serum (Gibco, Waltham, MA, USA) and 1% penicillin-streptomycin solution (Solarbio Science and Technology). Cells were grown in an air atmosphere with 5% (v/v) CO_2_ at 37°C.

### Lentiviral infection and establishment of USP44-stable cell lines

The full-length human *USP44* gene was cloned into GV492 vector (GeneChem, Westmount, QC, Canada). Using Lipofectamine 2000 transfection reagent (Thermo Fisher Scientific), HEK293FT cells were transfected with packaging plasmids pCMV-VSV-G, pRSV-Rev, and pMDLg/pRRE along with the USP44–DEST vector or control vector. At 48 h post-transfection, the viral supernatant was precipitated with PEG-it Virus Precipitation Solution (System Biosciences, Palo Alto, CA, USA) in a 1:4 ratio to produce a concentrated viral stock. HepG2 cells were plated 1 day before transduction. At 72 h post transduction, cells were selected in medium containing 2 μg/mL puromycin for 10 days. Cell lines stably expressing *USP44* or control vector were used for the functional study.

### Cell proliferation assays

Stably transfected HCC cells were seeded into a 96-well plate at a cell density of 4 × 10^3^ cells per well, with four replicate wells. The cells were incubated for 1–6 days. After incubation, cell proliferation was measured using the CCK-8 assay (Data Inventory Biotechnology, Hong Kong). The colorimetric product formed was measured at an absorbance of 450 nm and 600 nm (optical density change: OD_450 nm_ − OD_600 nm_).

### Cell cycle analysis by propidium iodide

Stably transfected cells (8 × 10^5^) were harvested when they reached approximately 80% confluence. The cells were trypsinized, washed with phosphate-buffered saline, and fixed in 1 mL ice-cold 70% ethanol at 4°C overnight. The fixed cells were pelleted by centrifugation at 1000 *g* for 5 min. The cells were then washed with phosphate-buffered saline and stained with 0.5 mL staining solution containing 50 μg/mL propidium iodide and 0.5 μg/mL RNase A for 30 min. The cells then underwent flow cytometric analysis using the FACSCanto and CellQuest software applications (BD Biosciences, Franklin Lakes, NJ, USA). Average values of the G0/G1, S, and G2/M phases were obtained from at least three independent experiments.

### Colony formation assay

Stably transfected HCC cells were seeded into 6-well plates (1000 cells/well). After incubation for 10 days, the cells were fixed in 4% paraformaldehyde for 30 min, stained with 0.1% crystal violet stain solution for 30 min, and then destained with water. The colonies (>20 cells/colony) were counted and recorded.

### Transcriptome sequencing and bioinformatic analysis

RNA quality and quantity were assessed using a Bioanalyzer 2100 and the RNA 6000 Nano LabChip Kit (Agilent Technologies, Santa Clara, CA, USA), and high-quality samples with an RNA integrity number >7.0 were used to construct the sequencing library. The cDNA library was ligated with indexed adapters, and the ligated products were amplified by PCR. The average insert size of the final cDNA library was approximately 300 bp. Next, 2 × 150 bp paired-end sequencing was performed on a NovaSeq system (Illumina, San Diego, CA, USA). High-quality clean reads were filtered using Cutadapt and the quality-trimmed sequence reads were mapped to the human genome reference (Homo sapiens Ensembl v96) using the HISAT2 software application (version: hisat2-2.0.4) [17]. The mapped reads of each sample were assembled using StringTie (version: stringtie-1.3.4d.Linux_x86_64) with default parameters [18]. StringTie and ballgown were used to estimate the expression levels of all transcripts and all genes. Genes with a |log2 (fold change: USP44/vector)| > 1 and log10 (*q* value) > 1.3 were considered to be differentially expressed genes (DEGs). The DEGs were subjected to gene ontology and pathway enrichment analysis using the Database for Annotation, Visualization, and Integrated Discovery tool [19]. Ingenuity Pathway Analysis (IPA) was used to further understand the diseases, biologic functions, and gene networks controlled by USP44-mediated DEGs.

### Statistical analysis

Statistical analyses of patient information, including age, sex, expression level of AFP, and the staging of HCC were performed using the Pearson chi-squared test. The positive of HBsAg in HCC, number of tumor nodules, degrees of differentiation, venous infiltration, and the presence of cirrhosis in HCC were performed using the Fisher's exact test. Size of largest tumor-length was assessed by the Student's *t*-test. The SPSS-PASW Statistics 18 software was used for statistical analyses. Correlations between clinicopathologic characteristics and *USP44* expression levels were assessed by the Student’s *t*-test to establish differential gene expression in relation to specific tumor phenotypes. Quantitative data are reported as means with standard deviation or standard error of the mean. The Wilcoxon matched-pairs signed rank test was used to compare *USP44* expression levels between groups. Patient survival was calculated using the Kaplan–Meier method from the date of surgery to the date of death or last follow-up. The log-rank test was used to compare survival between the groups. The Z-score is determined by using the Ingenuity Pathway Analysis. The DEGs of each treatment group were uploaded to the IPA system for functional analysis (Qiagen, Hilden, Germany). Canonical Pathway Analysis of IPA was used to identify significantly enriched canonical pathways in each treatment group, and Diseases and Functions Analysis of IPA was used to identify significantly enriched diseases and biological functions in each treatment group. The pathway or function with *P*-value < 0.05 was considered statistically significant. The activation z-score is to infer the activation states of predicted transcriptional regulators. The activation state of an upstream regulator is determined by the regulation direction associated with the relationship from the regulator to the gene.

### Availability of data and materials

Transcriptome sequencing data from this study have been deposited in the National Center for Biotechnology Information BioProject database (https://www.ncbi.nlm.nih.gov/bioproject) with the accession code PRJNA791131.

## RESULTS

### Low expression of USP44 is prognostic for HCC

**Figure 1:**
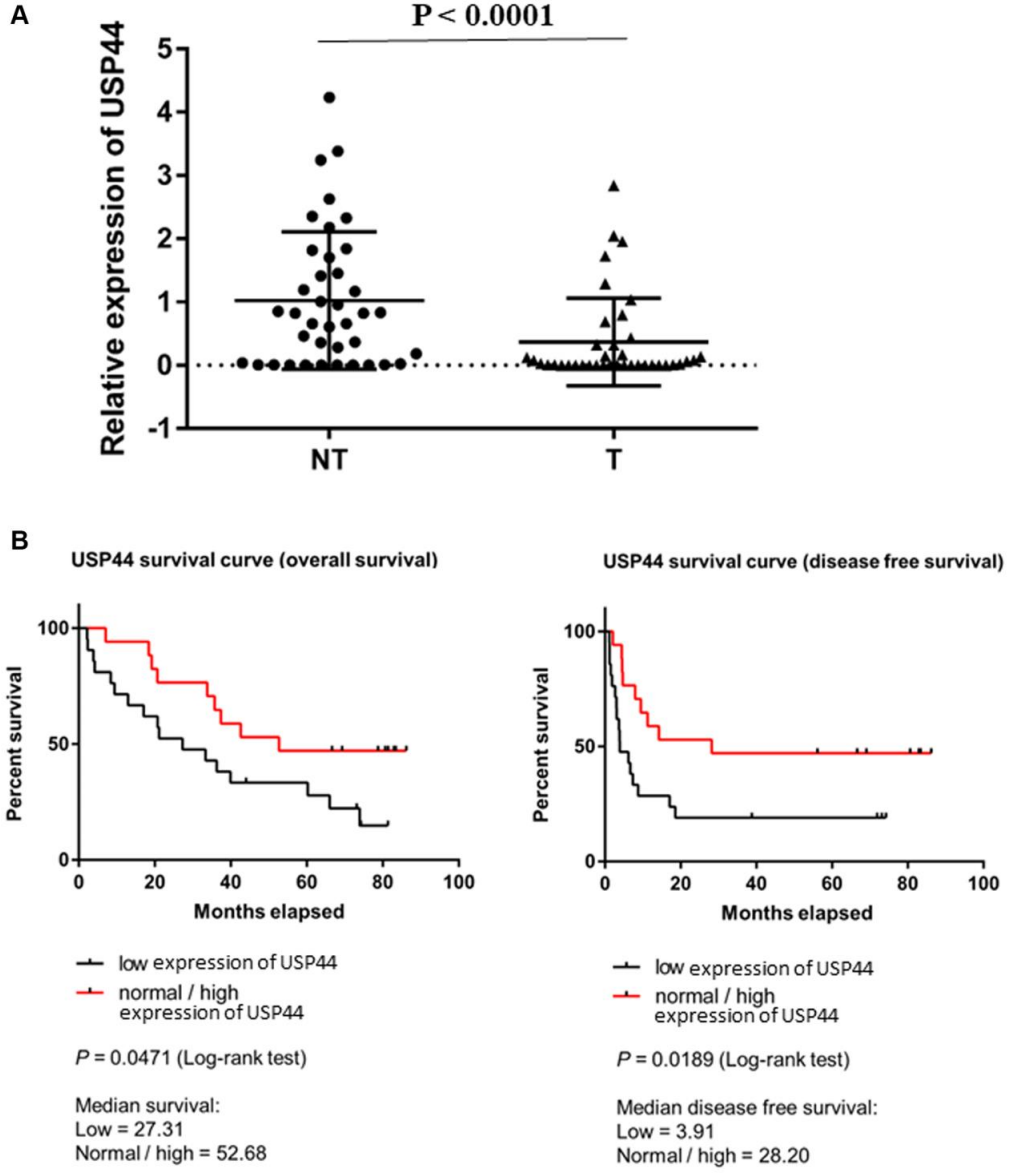
**Low expression of *USP44* is a prognostic factor in hepatocellular carcinoma (HCC).** (**A**) *USP44* expression in 40 pairs of HCC nontumoral (NT) and tumoral (T) tissues. *USP44* expression was lower in tumoral tissues than in nontumoral tissues (*p* < 0.0001). (**B**) Correlation between *USP44* expression and survival rate of patients with HCC. Low *USP44* expression was related to shorter overall survival (*p* = 0.047) and disease-free survival (*p* = 0.019) in patients with HCC. The *x*-axis indicates survival time in months. The *y*-axis indicates survival probability. The log-rank *p* < 0.05 indicates that *USP44* expression is significantly associated with prognosis in HCC.

We began by examining *USP44* expression in human HCC by analyzing 40 HCC tumors (T) and paired adjacent nontumoral liver tissue (NT). The quantitative RT-PCR results demonstrated significant downregulation of *USP44* in HCC tumors (57.5%) compared with adjacent nonmalignant liver tissue (*p* < 0.0001, Figure 1A), indicating that USP44 is commonly reduced in HCC. We next conducted a correlative analysis of the *USP44* expression in HCC tumors and the clinicopathologic features of the patients. The results showed that expression of *USP44* was lower in advanced-stage (stages IV) than in early-stage (stages II and III) HCC tumors (*p* = 0.049, Table 1), suggesting that *USP44* expression is related to tumor staging. In addition, overall survival (*p* = 0.0471) and disease-free survival (*p* = 0.0189) were significantly poorer in the low *USP44* expression group (median overall survival: 27.31 months; median disease-free survival: 3.98 months) than in the normal and high *USP44* expression groups (median overall survival: 52.68 months; median disease-free survival: 28.2 months; Figure 1B). Taken together, our results indicate that low *USP44* expression is a prognostic factor in HCC.

**Table 1:** Correlation between tumor USP44 expression and clinicopathologic features.

**Clinicopathological features**	**Frequency (%)**	**USP44 expression**		*P* value^*^
		**Mean ± SD/Number of cases**		
		**Low**	**Normal and High**	
**Age (year)**				
<59	18 (45.00%)	10	8	0.822
≥59	22 (55.00%)	13	9	
**Sex**				
Male	28 (70.00%)	18	10	0.185
Female	12 (30.00%)	5	7	
**HBsAg**				
Positive	32 (80.00%)	19	13	0.702
Negative	8 (20.00%)	4	4	
Number of tumor nodules^**^				
1	23 (60.53%)	11	12	0.182
≥2	15 (39.47%)	11	4	
**Differentiation**				
Well differentiated	6 (15.00%)	2	4	0.297
Moderately differentiated	23 (57.50%)	14	9	
Poorly differentiated	10 (25.00%)	7	3	
Undifferentiated	1(2.50%)	0	1	
Venous infiltration^**^				
Absence	15 (39.47%)	8	7	1.000
Presence	23 (60.53%)	13	10	
Non-tumor liver^**^				
Chronic hepatitis	7 (17.95%)	4	3	0.248
Cirrhotic	25 (64.10%)	12	13	
Non-cirrhotic	7 (17.95%)	6	1	
**TNM**				
II and III	21 (52.50%)	9	12	0.049
IV	19 (47.50%)	14	5	
**AFP (ng/ml)**				
<400	25 (62.50%)	16	9	0.283
≥400	15 (37.50%)	7	8	
Size of largest tumor-length (cm)^**^		12.18 ± 4.53	9.09 ± 5.52	0.066

^*^Pearson Chip-square or fisher’s exact test for number of cases, *t*-test for mean. ^**^Total number less than 40 due to missing data.

### Overexpression of USP44 is associated with reduced proliferation in an HCC cell line

**Figure 2:**
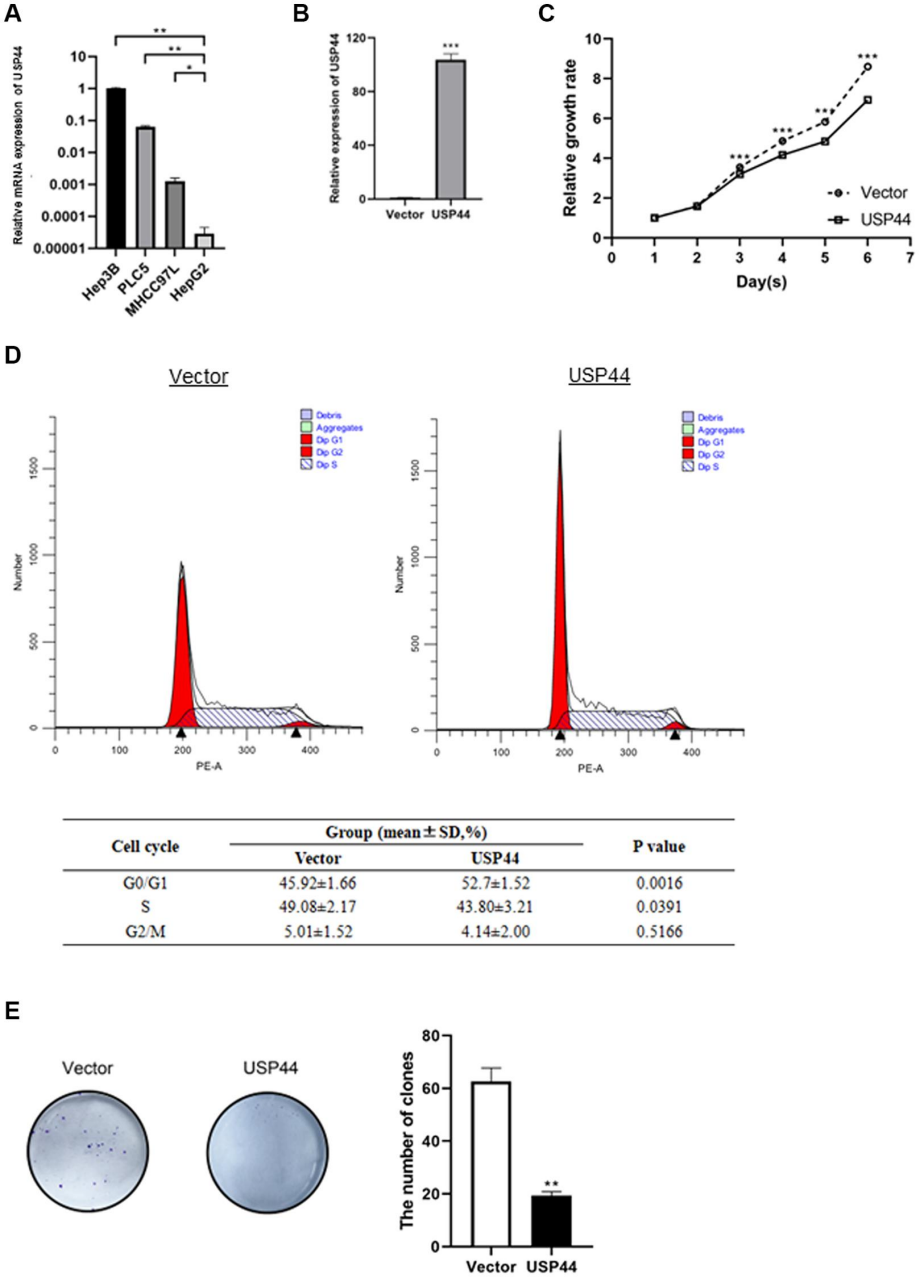
**Roles of USP44 in cell proliferation and the cell cycle in hepatocellular carcinoma (HCC).** (**A**) *USP44* expression in various HCC cell lines, including Hep3B, PLC5, MHCC97L, and HepG2. *USP44* expression was relatively lower in the HepG2 cell line than in other HCC cell lines. (**B**) Quantitative real-time PCR analysis validated the overexpression of *USP44* after lentiviral infection with either USP44 vector or control vector. (**C**) The CCK-8 assay showed inhibition of HepG2 cell proliferation after *USP44* overexpression. Dashed line = control vector HepG2 cells; solid line = *USP44*-overexpressing HepG2 cells. (**D**) Flow cytometry analysis with propidium iodide staining shows cell cycle arrest at the G0/G1 phase in *USP44*-overexpressing HepG2 cells. (**E**) *USP44* overexpression was associated with reduced colony formation by HepG2 cells. Colony formation was assessed 10 days after 1000 cells were seeded onto a 6-well plate.

In a gain-of-function study, we used an HCC cell line model to determine the role of USP44 in the carcinogenicity of HCC. The HCC cell line HepG2 has relatively low *USP44* mRNA expression as compared to other HCC cell lines including Hep3B, PLC-5, and MHCC97L (Figure 2A). Overexpression of *USP44* in HepG2 cells was validated at the gene levels by quantitative RT-PCR (Figure 2B). We next investigated the functional role of USP44 in HCC. Our results showed that the overexpression of *USP44* could significantly inhibit the growth of HCC cells (Figure 2C) and arrest the cell cycle at the G0/G1 phase (Figure 2D). In addition, *USP44* overexpression reduced the ability of HCC cells to form colonies (Figure 2E). Collectively, these data suggest the importance of USP44 in HCC tumorigenicity.

### USP44 controlled the processes and functions related to HCC carcinogenesis and metastasis

**Figure 3:**
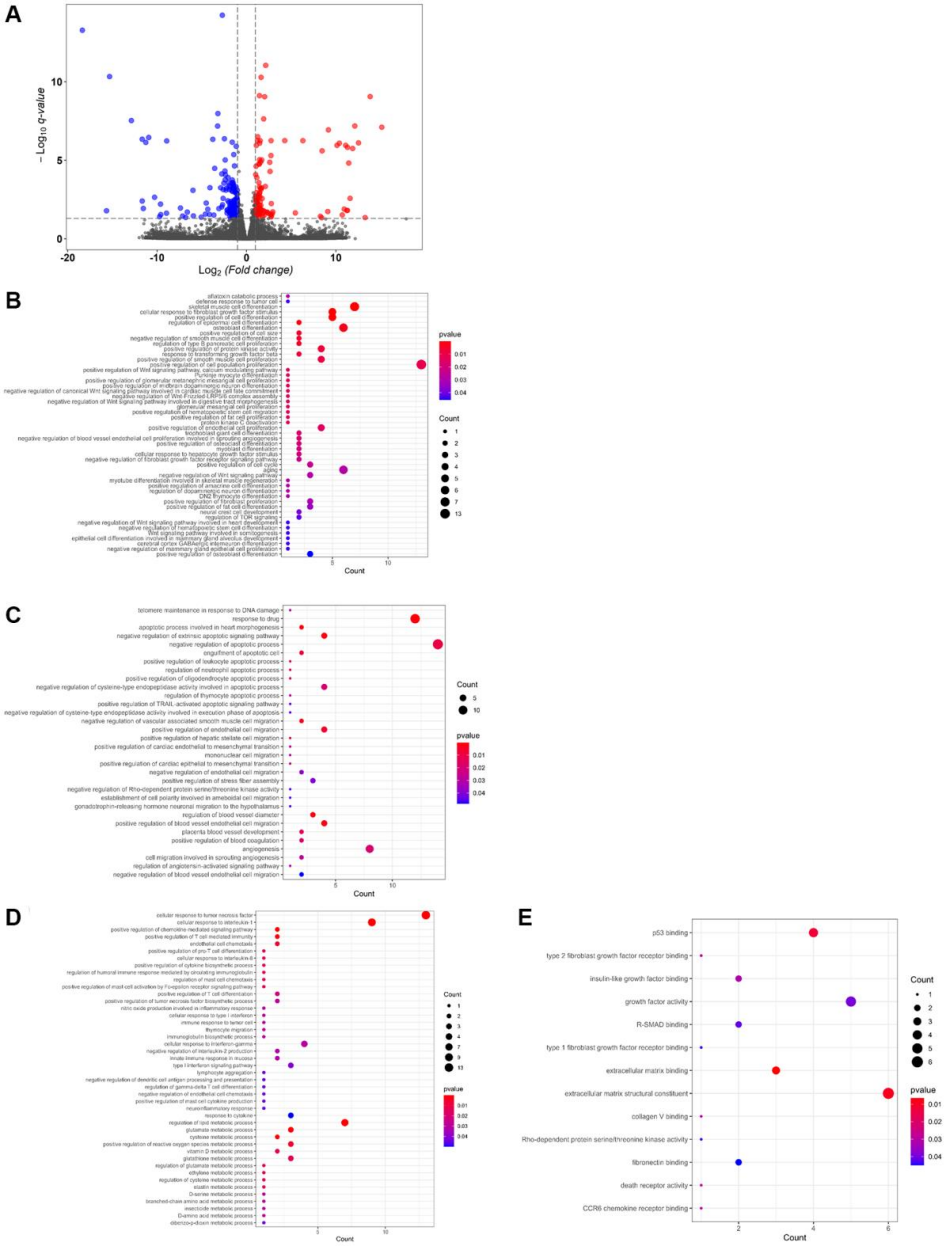
**Differential gene expression controlled by USP44 in hepatocellular carcinoma (HCC) cells.** (**A**) A volcano plot shows differential gene expression associated with *USP44* overexpression in HepG2 cell. Blue dots = downregulated genes; red dots = upregulated genes; and grey dots = unchanged genes. The *x*-axis represents differential gene expression in a log2 ratio for the *USP44*-overexpressing cells compared with the control vector cells. The *y*-axis represents the *q* value for differential gene expression, comparing *USP44*-overexpressing cells with control vector cells. (**B**) Gene ontology enrichment analysis of USP44-mediated differentially expressed genes highlights the biologic processes related to cell proliferation and differentiation. Bubble diagrams show the biologic processes related (**C**) to DNA damage response, drug response, cell apoptosis, metastasis, and angiogenesis; (**D**) to the immune response and metabolism; and (**E**) to the binding and kinase activities related to cell growth, metastasis, and immune response controlled by USP44 in HCC cells. The size of the bubbles reflects the number of genes being controlled. The color of the bubble reflects the significance of the processes being controlled.

To delineate the molecular mechanisms underlying the role of USP44 in HCC carcinogenicity, a comparative transcriptomic analysis of *USP44*-overexpressing HepG2 cells and control vector–expressing HepG2 cells was conducted. We obtained at least 40 million qualified sequencing reads from each transcriptome sequencing sample, for a sequencing data total of 51.22 Gb (Table 2). The average mapping to the human genome exceeded 95%. When we compared the transcriptome profiles of *USP44*-overexpressing HepG2 cells and control cells, we identified 292 DEGs, including 115 upregulated and 177 downregulated genes (Figure 3A and Supplementary Table 1). Gene ontology enrichment analysis highlighted the importance of those genes in biologic processes related to cell proliferation and differentiation through the regulation of cell signaling such as Wnt signaling, TOR signaling, and protein kinase C signaling (Figure 3B). In addition, the USP44-mediated genes were found to control the DNA damage response, drug response, cell apoptosis via the regulation of TRAIL (Figure 3C). Furthermore, the targeted genes play important roles in metastasis and angiogenesis through the regulation of cell migration ability and angiotensin-activated signaling (Figure 3C). More importantly, USP44 could control the immune response via the regulation of T cell and production of interleukins, and mediate different metabolisms such as metabolisms of vitamin D, lipid, reactive oxygen species, and glutathione (Figure 3D). In the analysis of molecular function, we also observed many binding and kinase activities related to cell growth, metastasis, and immune response, including p53 binding, type 2 fibroblast growth factor receptor binding, insulin-like growth factor binding, and death receptor activity (Figure 3E). Taken together, our data suggest that USP44 controls a cluster of genes related to HCC carcinogenicity by modulating cell proliferation, apoptosis, metastasis, and immune functions.

**Table 2:** Quality Check of transcriptome sequencing data.

**Sample**	**Raw data**		**Valid data**		**Valid ratio (reads)**	**Q20 (%)**	**Q30 (%)**	**GC content (%)**	**Mapped reads**	**Mapped rate (%)**
	**Read**	**Base (Gb)**	**Read**	**Base (Gb)**						
**Control vector_01**	45575002	6.84	43997364	6.60	96.54	99.95	97.97	49.50	42138167	95.77
**Control vector_02**	45164274	6.77	43358110	6.50	96.00	99.95	97.90	49.50	41451356	95.60
**Control vector_03**	42840724	6.43	41194828	6.18	96.16	99.95	98.00	49.50	39481705	95.84
**Control vector_04**	45939612	6.89	44361892	6.65	96.57	99.95	97.91	51.50	42486946	95.77
**USP44_01**	42496338	6.37	40973924	6.15	96.42	99.94	97.89	52.50	39179302	95.62
**USP44_02**	44802690	6.72	43216644	6.48	96.46	99.95	97.97	50.50	41294695	95.55
**USP44_03**	45018236	6.75	43174270	6.48	95.90	99.95	98.02	50.00	41326554	95.72
**USP44_04**	43067850	6.46	41221908	6.18	95.71	99.95	97.92	50.50	39460418	95.73

### Overexpression of USP44 induces biologic functions and pathways related to DNA damage response and cell apoptosis in HCC

**Figure 4:**
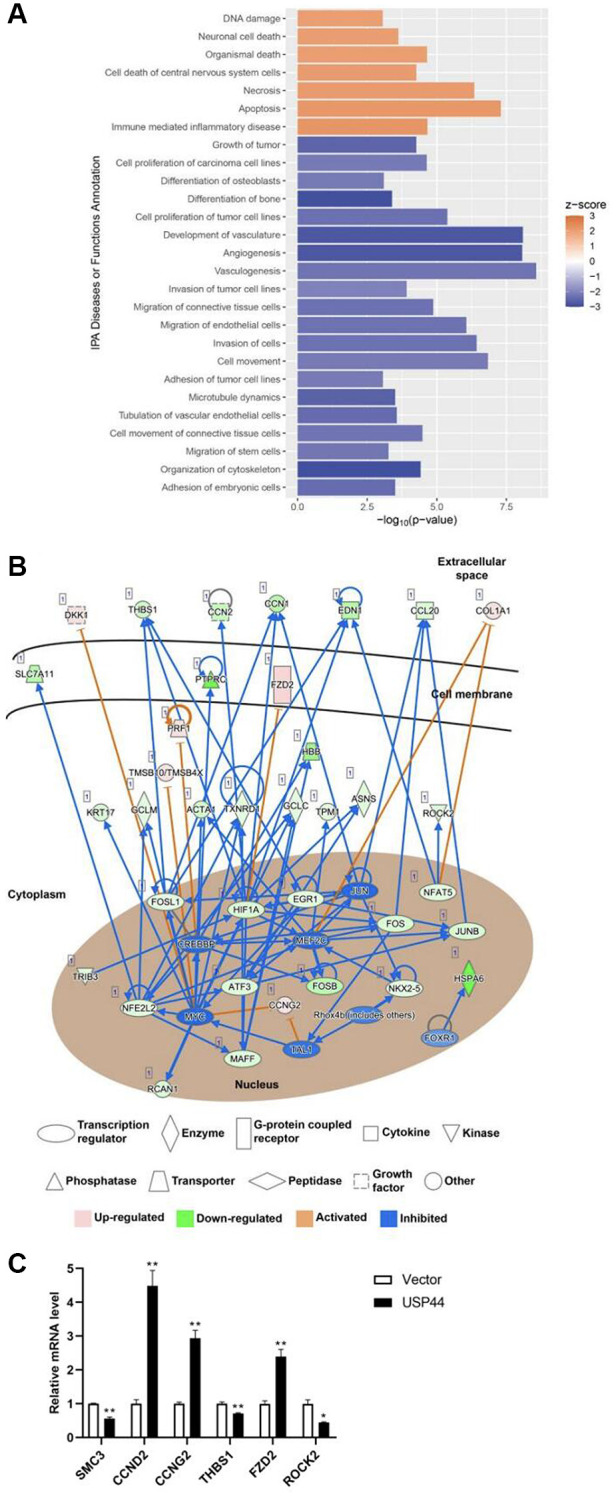
***USP44* overexpression reduced proliferation and induced apoptosis in hepatocellular carcinoma (HCC) cells.** (**A**) Ingenuity Pathway Analysis (IPA) demonstrated induction of apoptosis-related and reduction of cell proliferation-related diseases and functions caused by *USP44* overexpression in HepG2 HCC cells. A positive z score represents activation; a negative z score represents inhibition. (**B**) IPA gene network construction shows how the functional role of USP44 in HCC involves receptors, enzymes, cytokines, kinases, phosphatases, and transcription factors. Red diagrams = upregulated genes; green diagrams = downregulated genes; orange arrows = activation; and blue arrows = inhibition. (**C**) qPCR analysis was used to validate the differential gene expression obtained from transcriptome sequencing.

In the IPA conducted to further understand the role of USP44 and its controlled gene networks, we focused on the diseases and biologic functions and the canonical pathways controlled by USP44 in HCC. The results showed that *USP44* overexpression could induce biologic functions related to DNA damage, cell apoptosis, and necrosis in HCC (Figure 4A), which is reflected in a positive Z-score. In contrast, *USP44* overexpression reduced the proliferation and migration ability of tumor cells in HCC (Figure 4A). In addition, induction of USP44 led to the inhibition of angiogenesis and vasculogenesis in HCC cells (Figure 4A).

In the IPA, we further investigated the network of canonical pathways controlled by USP44 in HCC. Our results showed the involvement of cytokines such as DKK1, CCN2, EDN1, JAG1, and CCL20 in the functional roles of USP44 in HCC (Figure 4B). The cytokines targeted membrane proteins and receptors including KTN1, FZD2, NGFR, and SFRP5, leading to the control of enzymes such as PLA2G4B, RAC2, RASD1, GCLM, GCLC, ASNS, CYP1A1, and TAT (Figure 4B). In addition, USP44 mediated many transcription factors and cyclins involved in the control of cell proliferation, metastasis, and apoptosis in HCC—for example, NFAT5, EGR1, FOSL1, NFE2L2, NKX2-5, HIF1A, FOS, JUNB, MAFF, SIRT1, BRAC2, FOSB, ATF3, CEBPZ, MEF2B, CCND2, and CCNG2 (Figure 4B). The result of transcriptome was validated by using qPCR, and we found that the results of transcriptome sequencing and qPCR were well-matched (Figure 4C).

## DISCUSSION

To date, few studies have investigated the correlation between USP44 and HCC. In the present study, we investigated the prognostic value of USP44 in HCC. By comparing *USP44* expression levels in 40 pairs of HCC tumoral and nontumoral tissues, we found a significant reduction in *USP44* expression in the tumoral tissues. More importantly, low *USP44* expression was associated with advanced-stage HCC and poorer patient survival rates in HCC. This finding is similar to those described in previous reports about pancreatic and colorectal cancers, in which *USP44* was found to be downregulated in patients with pancreatic ductal adenocarcinoma and colorectal cancer [20, 21], suggesting that USP44 is a prognostic marker in patients with cancer. Given that the role of USP44 in the carcinogenicity of HCC is still unknown, we used a gain-of-function analysis to characterize USP44 in HCC HepG2 cells. Our results demonstrated that *USP44* overexpression reduced HCC proliferation by arresting the cell cycle at G0/G1. In addition, induction of USP44 inhibited the colony-forming ability of HCC cells. These results accord with many clinical studies showing that USP44 is a tumor suppressor that inhibits cancer-cell growth [15]. *In vivo* and *in vitro* studies have both shown that USP44 suppresses tumorigenesis in nasopharyngeal carcinoma by regulating DNA damage repair [22]. USP44 was also reported to inhibit cell proliferation in colorectal cancer [21], clear cell renal cell carcinoma [23], and non–small cell lung cancer [24]. However, contradictory findings have been reported in gastric and prostate carcinomas, in which *USP44* upregulation was associated with promotion of cancer progression and tumorigenesis, suggesting that USP44 has various roles in various cancer types [25, 26]. After functional characterization, we investigated the targets and cell signaling pathways regulated by USP44 in HCC. Using comparative transcriptomic analysis, we identified 292 USP44 targets. Gene ontology enrichment analysis and IPA further highlighted the involvement of USP44-mediated genes in cell proliferation. Overexpression of USP44 led to upregulation of *CCND2* and *CCNG2* and downregulation of *SMC3*, which are cell-cycle-related genes. Cyclin D2 *(CCND2)* is a core component of the machinery that drives cell cycle progression; it is associated with tumorigenesis [27]. However, the evidence about the function of *CCND2* in tumorigenesis is contradictory, and some reports have suggested that *CCND2* is an oncogene. For instance, an increase in *CCND2* has been reported to promote cell growth in colorectal cancer [28]. A genome-wide CRISPR-Cas9 study also demonstrated the importance of *CCND2* in the proliferation and survival of adult T-cell leukemia/lymphoma cells [29]. However, a study looking at non–small cell lung cancer showed reduced expression of *CCND2*, and the level of *CCND2* was adversely associated with recurrence-free survival in patients with non–small cell lung cancer [30]. In addition, the methylation rate of the *CCND2* promoter was significantly higher in patients with HCC than in those without HCC. More importantly, advanced-stage HCC was associated with lower mRNA levels of *CCND2* [31].

Unlike other cyclins, which positively regulate the cell cycle, cyclin G2 (*CCNG2*) regulates cell proliferation as a tumor suppressor gene [32]. *CCNG2* is associated with various types of tumors and causes cell cycle arrest at the G0/G1 phase. *CCNG2* inhibits cell cycle progression in head and neck squamous cell carcinomas and Raji lymphoma cells [33, 34]. Further, deletion of *CCNG2* led to a decrease in cells in the G0/G1 phase in human colorectal cancer, and overexpression of *CCNG2* prevented G1/S phase transformation in colorectal cancer cells [16]. Structural maintenance of chromosomes protein 3 *(SMC3)*, a member of the chromosome structural maintenance family, helps to maintain chromosomal stability by forming heterodimers. *SMC3* is highly expressed in HCC tissues and is positively correlated with poor prognosis in affected patients [35].

In the IPA, we found that *USP44* controlled both frizzled family protein 2 *(FZD2)* and rho-associated protein kinase 2 *(ROCK2)*, which are associated with the Wnt signaling pathway. The FZDs (including FZD1–FZD10) function as cell surface receptors in that pathway. Each member of the FZDs can activate Wnt signaling by interacting with various Wnt proteins [36]. Deletion of *FZD2* inhibited the migration and invasiveness of HCC cells but did not affect HCC cell proliferation [37]. *FZD2* overexpression was able to activate Wnt/non-canonical planar cell polarity in the HCC cell membrane signaling pathway to mediate HCC invasion and metastasis [38]. *ROCK2* is a cytoskeletal regulator. It has been reported that *ROCK2* mRNA and protein expression levels are significantly higher in HCC and that the *ROCK2* rs9808232 polymorphism promoted its expression in liver tissue, thereby increasing susceptibility to, and poor prognosis in, HCC [39]. Thus, overexpression of *USP44*-reduced *ROCK2* might reduce HCC tumorigenicity.

In the present report, we focused mainly on the role of USP44 in HCC cell proliferation through the regulation of gene transcription. However, the findings were mainly from one single cell line. Further study needed to be carried out to specify the function of USP44 by experiments knocking down/depletion of in Hep2G overexpressing USP44 to see whether it would increase malignant phenotype in the cells. In addition, further clinical studies are needed to confirm our findings. USP44 is a deubiquitinating enzyme that controls its targets post-transcriptionally through deubiquitination. Thus, further studies on the deubiquitination targets of USP44 could provide novel insights into the molecular mechanisms controlled by USP44 in HCC. Moreover, USP44 has been reported to regulate various functions, such as metastasis and DNA damage response in other cancer types [23]. The latter functions could be one of the directions to pursue during further investigation of the role of USP44 in HCC. To summarize, our study has provided novel insights into the prognostic value of USP44 in HCC and provides evidence that USP44 could be a possible therapeutic target in HCC.

## Supplementary Materials

Supplementary Table 1

## References

[1] Sung H, Ferlay J, Siegel RL, Laversanne M, Soerjomataram I, Jemal A, Bray F. Global Cancer Statistics 2020: GLOBOCAN Estimates of Incidence and Mortality Worldwide for 36 Cancers in 185 Countries. CA Cancer J Clin. 2021; 71:209–49. 10.3322/caac.21660 33538338

[2] Yang SF, Chang CW, Wei RJ, Shiue YL, Wang SN, Yeh YT. Involvement of DNA damage response pathways in hepatocellular carcinoma. Biomed Res Int. 2014; 2014:153867. 10.1155/2014/153867 24877058 PMC4022277

[3] Xie X, Hu H, Tong X, Li L, Liu X, Chen M, Yuan H, Xie X, Li Q, Zhang Y, Ouyang H, Wei M, Huang J, et al. The mTOR-S6K pathway links growth signalling to DNA damage response by targeting RNF168. Nat Cell Biol. 2018; 20:320–31. 10.1038/s41556-017-0033-8 29403037 PMC5826806

[4] Wang M, Sun Y, Xu J, Lu J, Wang K, Yang DR, Yang G, Li G, Chang C. Preclinical studies using miR-32-5p to suppress clear cell renal cell carcinoma metastasis via altering the miR-32-5p/TR4/HGF/Met signaling. Int J Cancer. 2018; 143:100–12. 10.1002/ijc.31289 29396852 PMC5938119

[5] Li G, Liu D, Kimchi ET, Kaifi JT, Qi X, Manjunath Y, Liu X, Deering T, Avella DM, Fox T, Rockey DC, Schell TD, Kester M, Staveley-O'Carroll KF. Nanoliposome C6-Ceramide Increases the Anti-tumor Immune Response and Slows Growth of Liver Tumors in Mice. Gastroenterology. 2018; 154:1024–36.e9. 10.1053/j.gastro.2017.10.050 29408569 PMC5908238

[6] Kauffmann A, Rosselli F, Lazar V, Winnepenninckx V, Mansuet-Lupo A, Dessen P, van den Oord JJ, Spatz A, Sarasin A. High expression of DNA repair pathways is associated with metastasis in melanoma patients. Oncogene. 2008; 27:565–73. 10.1038/sj.onc.1210700 17891185

[7] Hanpude P, Bhattacharya S, Dey AK, Maiti TK. Deubiquitinating enzymes in cellular signaling and disease regulation. IUBMB Life. 2015; 67:544–55. 10.1002/iub.1402 26178252

[8] Agathanggelou A, Smith E, Davies NJ, Kwok M, Zlatanou A, Oldreive CE, Mao J, Da Costa D, Yadollahi S, Perry T, Kearns P, Skowronska A, Yates E, et al. USP7 inhibition alters homologous recombination repair and targets CLL cells independently of ATM/p53 functional status. Blood. 2017; 130:156–66. 10.1182/blood-2016-12-758219 28495793

[9] Hernández-Pérez S, Cabrera E, Salido E, Lim M, Reid L, Lakhani SR, Khanna KK, Saunus JM, Freire R. DUB3 and USP7 de-ubiquitinating enzymes control replication inhibitor Geminin: molecular characterization and associations with breast cancer. Oncogene. 2017; 36:4802–9. 10.1038/onc.2017.21 28288134

[10] Sloane MA, Wong JW, Perera D, Nunez AC, Pimanda JE, Hawkins NJ, Sieber OM, Bourke MJ, Hesson LB, Ward RL. Epigenetic inactivation of the candidate tumor suppressor USP44 is a frequent and early event in colorectal neoplasia. Epigenetics. 2014; 9:1092–100. 10.4161/epi.29222 24837038 PMC4164494

[11] Zhang Y, van Deursen J, Galardy PJ. Overexpression of ubiquitin specific protease 44 (USP44) induces chromosomal instability and is frequently observed in human T-cell leukemia. PLoS One. 2011; 6:e23389. 10.1371/journal.pone.0023389 21853124 PMC3154946

[12] Zou Y, Qiu G, Jiang L, Cai Z, Sun W, Hu H, Lu C, Jin W, Hu G. Overexpression of ubiquitin specific proteases 44 promotes the malignancy of glioma by stabilizing tumor-promoter securin. Oncotarget. 2017; 8:58231–46. 10.18632/oncotarget.16447 28938551 PMC5601647

[13] Zhang W, Sulea T, Tao L, Cui Q, Purisima EO, Vongsamphanh R, Lachance P, Lytvyn V, Qi H, Li Y, Ménard R. Contribution of active site residues to substrate hydrolysis by USP2: insights into catalysis by ubiquitin specific proteases. Biochemistry. 2011; 50:4775–85. 10.1021/bi101958h 21542621

[14] Cheng J, Demeulemeester J, Wedge DC, Vollan HKM, Pitt JJ, Russnes HG, Pandey BP, Nilsen G, Nord S, Bignell GR, White KP, Børresen-Dale AL, Campbell PJ, et al. Author Correction: Pan-cancer analysis of homozygous deletions in primary tumours uncovers rare tumour suppressors. Nat Commun. 2019; 10:525. 10.1038/s41467-019-08512-7 30692535 PMC6349916

[15] Holland AJ, Cleveland DW. The deubiquitinase USP44 is a tumor suppressor that protects against chromosome missegregation. J Clin Invest. 2012; 122:4325–8. 10.1172/JCI66420 23187131 PMC3533566

[16] Liu T, Sun B, Zhao X, Li Y, Zhao X, Liu Y, Yao Z, Gu Q, Dong X, Shao B, Lin X, Liu F, An J. USP44+ Cancer Stem Cell Subclones Contribute to Breast Cancer Aggressiveness by Promoting Vasculogenic Mimicry. Mol Cancer Ther. 2015; 14:2121–31. 10.1158/1535-7163.MCT-15-0114-T 26232424

[17] Kim D, Paggi JM, Park C, Bennett C, Salzberg SL. Graph-based genome alignment and genotyping with HISAT2 and HISAT-genotype. Nat Biotechnol. 2019; 37:907–15. 10.1038/s41587-019-0201-4 31375807 PMC7605509

[18] Pertea M, Kim D, Pertea GM, Leek JT, Salzberg SL. Transcript-level expression analysis of RNA-seq experiments with HISAT, StringTie and Ballgown. Nat Protoc. 2016; 11:1650–67. 10.1038/nprot.2016.095 27560171 PMC5032908

[19] Huang da W, Sherman BT, Lempicki RA. Bioinformatics enrichment tools: paths toward the comprehensive functional analysis of large gene lists. Nucleic Acids Res. 2009; 37:1–13. 10.1093/nar/gkn923 19033363 PMC2615629

[20] Yang C, Zhu S, Yang H, Deng S, Fan P, Li M, Jin X. USP44 suppresses pancreatic cancer progression and overcomes gemcitabine resistance by deubiquitinating FBP1. Am J Cancer Res. 2019; 9:1722–33. 31497353 PMC6726996

[21] Huang T, Zhang Q, Ren W, Yan B, Yi L, Tang T, Lin H, Zhang Y. USP44 suppresses proliferation and enhances apoptosis in colorectal cancer cells by inactivating the Wnt/β-catenin pathway via Axin1 deubiquitination. Cell Biol Int. 2020; 44:1651–9. 10.1002/cbin.11358 32285989 PMC7496820

[22] Chen Y, Zhao Y, Yang X, Ren X, Huang S, Gong S, Tan X, Li J, He S, Li Y, Hong X, Li Q, Ding C, et al. USP44 regulates irradiation-induced DNA double-strand break repair and suppresses tumorigenesis in nasopharyngeal carcinoma. Nat Commun. 2022; 13:501. 10.1038/s41467-022-28158-2 35079021 PMC8789930

[23] Zhou J, Wang T, Qiu T, Chen Z, Ma X, Zhang L, Zou J. Ubiquitin-specific protease-44 inhibits the proliferation and migration of cells via inhibition of JNK pathway in clear cell renal cell carcinoma. BMC Cancer. 2020; 20:214. 10.1186/s12885-020-6713-y 32164618 PMC7068999

[24] Zhang YK, Tian WZ, Zhang RS, Zhang YJ, Ma HT. Ubiquitin-specific protease 44 inhibits cell growth by suppressing AKT signaling in non-small cell lung cancer. Kaohsiung J Med Sci. 2019; 35:535–41. 10.1002/kjm2.12096 31197957 PMC11900763

[25] Xiang T, Jiang HS, Zhang BT, Liu G. CircFOXO3 functions as a molecular sponge for miR-143-3p to promote the progression of gastric carcinoma via upregulating USP44. Gene. 2020; 753:144798. 10.1016/j.gene.2020.144798 32445925

[26] Park JM, Lee JE, Park CM, Kim JH. USP44 Promotes the Tumorigenesis of Prostate Cancer Cells through EZH2 Protein Stabilization. Mol Cells. 2019; 42:17–27. 10.14348/molcells.2018.0329 30622230 PMC6354053

[27] Ding ZY, Li R, Zhang QJ, Wang Y, Jiang Y, Meng QY, Xi QL, Wu GH. Prognostic role of cyclin D2/D3 in multiple human malignant neoplasms: A systematic review and meta-analysis. Cancer Med. 2019; 8:2717–29. 10.1002/cam4.2152 30950241 PMC6558476

[28] Zhang W, Wang B, Lin Y, Yang Y, Zhang Z, Wang Q, Zhang H, Jiang K, Ye Y, Wang S, Shen Z. hsa_circ_0000231 Promotes colorectal cancer cell growth through upregulation of CCND2 by IGF2BP3/miR-375 dual pathway. Cancer Cell Int. 2022; 22:27. 10.1186/s12935-022-02455-8 35033075 PMC8760675

[29] Ishio T, Kumar S, Shimono J, Daenthanasanmak A, Dubois S, Lin Y, Bryant B, Petrus MN, Bachy E, Huang DW, Yang Y, Green PL, Hasegawa H, et al. Genome-wide CRISPR screen identifies CDK6 as a therapeutic target in adult T-cell leukemia/lymphoma. Blood. 2022; 139:1541–56. 10.1182/blood.2021012734 34818414 PMC8914179

[30] Ko E, Kim Y, Park SE, Cho EY, Han J, Shim YM, Park J, Kim DH. Reduced expression of cyclin D2 is associated with poor recurrence-free survival independent of cyclin D1 in stage III non-small cell lung cancer. Lung Cancer. 2012; 77:401–6. 10.1016/j.lungcan.2012.03.027 22534667

[31] Qian Y, Wang JW, Fang Y, Yuan XD, Fan YC, Gao S, Wang K. Measurement of Cyclin D2 (CCND2) Gene Promoter Methylation in Plasma and Peripheral Blood Mononuclear Cells and Alpha-Fetoprotein Levels in Patients with Hepatitis B Virus-Associated Hepatocellular Carcinoma. Med Sci Monit. 2020; 26:e927444. 10.12659/MSM.927444 33320844 PMC7749526

[32] Hasegawa S, Nagano H, Konno M, Eguchi H, Tomokuni A, Tomimaru Y, Wada H, Hama N, Kawamoto K, Kobayashi S, Marubashi S, Nishida N, Koseki J, et al. Cyclin G2: A novel independent prognostic marker in pancreatic cancer. Oncol Lett. 2015; 10:2986–90. 10.3892/ol.2015.3667 26722276 PMC4665725

[33] Huang Q, Shen YJ, Hsueh CY, Guo Y, Zhang YF, Li JY, Zhou L. miR-17-5p drives G2/M-phase accumulation by directly targeting CCNG2 and is related to recurrence of head and neck squamous cell carcinoma. BMC Cancer. 2021; 21:1074. 10.1186/s12885-021-08812-6 34598688 PMC8487119

[34] Zheng C, Xiao Y, Li Y, He D. Knockdown of long non-coding RNA PVT1 inhibits the proliferation of Raji cells through cell cycle regulation. Oncol Lett. 2019; 18:1225–34. 10.3892/ol.2019.10450 31423183 PMC6607259

[35] Nie H, Wang Y, Yang X, Liao Z, He X, Zhou J, Ou C. Clinical Significance and Integrative Analysis of the SMC Family in Hepatocellular Carcinoma. Front Med (Lausanne). 2021; 8:727965. 10.3389/fmed.2021.727965 34527684 PMC8437102

[36] Zeng CM, Chen Z, Fu L. Frizzled Receptors as Potential Therapeutic Targets in Human Cancers. Int J Mol Sci. 2018; 19:1543. 10.3390/ijms19051543 29789460 PMC5983605

[37] Asano T, Yamada S, Fuchs BC, Takami H, Hayashi M, Sugimoto H, Fujii T, Tanabe KK, Kodera Y. Clinical implication of Frizzled 2 expression and its association with epithelial-to-mesenchymal transition in hepatocellular carcinoma. Int J Oncol. 2017; 50:1647–54. 10.3892/ijo.2017.3937 28350091

[38] Liu Y, Deng H, Liang L, Zhang G, Xia J, Ding K, Tang N, Wang K. Depletion of VPS35 attenuates metastasis of hepatocellular carcinoma by restraining the Wnt/PCP signaling pathway. Genes Dis. 2020; 8:232–40. 10.1016/j.gendis.2020.07.009 33997170 PMC8099696

[39] Qin L, Liu X, Lan L, Lv X. ROCK2 Polymorphism and Expression Contribute to Increased Susceptibility and Poor Prognosis in Hepatocellular Carcinoma. Int J Gen Med. 2022; 15:1295–306. 10.2147/IJGM.S343968 35173468 PMC8841339

